# What Counts for the Old and Oldest Old?—An Analysis of Patient Criteria for Choosing a Dentist—Part I: Awareness and Selection Criteria, Infrastructure, and Dental Office Equipment

**DOI:** 10.3390/ijerph19148307

**Published:** 2022-07-07

**Authors:** Ina Nitschke, Richard von Chlingensperg, Annett Schrock, Werner Hopfenmüller, Julia Jockusch

**Affiliations:** 1 Gerodontology Section, Department of Prosthodontics and Materials Science, University of Leipzig, Liebigstraße 12, 04103 Leipzig, Germany or ina.nitschke@zzm.uzh.ch (I.N.); richard.vonchlingensperg@medizin.uni-leipzig.de (R.v.C.); annett.schrock@medizin.uni-leipzig.de (A.S.); 2 Clinic of General, Special Care and Geriatric Dentistry, Center of Dental Medicine, University of Zurich, Plattenstrasse 11, 8032 Zurich, Switzerland; 3 Institute of Biometry and Clinical Epidemiology, Charité—Universitätsmedizin Berlin, Corporate Member of Freie Universität Berlin, Humboldt-Universität zu Berlin, and Berlin Institute of Health, 10117 Berlin, Germany; werner.hopfenmueller@charite.de; 4 University Research Priority Program “Dynamics of Healthy Aging”, University of Zurich, 8050 Zurich, Switzerland

**Keywords:** ideal dentist, patients’ perceptions, awareness, selection criteria, utilization, dental service uptake, influencing factors, infrastructure, dental office, telephone survey

## Abstract

Utilization of a dentist is influenced by many factors. The aim of this study is to present the factors relating to how patients become aware of a dentist, according to which criteria they select the dentist, and which factors in the infrastructure, equipment of dental offices, and human interactions are important for patients. A telephone survey with 466 participants (female 59.9%) in three age groups (ag 1: 35–50 years, ag 2: 70–84 years, ag 3: >85 years) in three German cities was conducted. Data were analyzed with respect to age, gender, and place of residence. Hardly any differences in the selection of the dentist and the selection criteria applied were found between the sexes, the age groups, or the places of residence. Recommendation seems to be the major aspect regarding how patients become aware of or select their dentist (*n* = 278, 65.6%), while modern technologies, e.g., the internet, play a subordinate role (*n* = 31, 7.3%). The unimportance of modern technologies increases significantly with the increase in age. As age increases, factors such as infrastructure (e.g., elevator available (ANOVA *p* < 0.001; Bonferoni correction: significant differences between ag 1 and ag 2 *p* < 0.001, ag 1 and ag 3 *p* < 0.001, and ag 2 and ag 3 *p* = 0.009); accessibility by wheelchair (ANOVA *p* < 0.001; Bonferoni correction: significant differences between ag 1 and ag 2 *p* = 0.006; and ag 1 and ag 3 *p* < 0.001); etc.) and dental office equipment become significantly important and influence the choice of dentist, while the importance of good parking facilities significantly decreased with age (ANOVA *p* = 0.003; Bonferoni correction: significant differences between ag 1 and ag 3 *p* = 0.004, and ag 2 and ag 3 *p* = 0.023). With increasing age, e.g., the importance of a television in the waiting room (ANOVA *p* = 0.012; Bonferoni correction: significant differences between ag 1 and ag 3 *p* = 0.014; and ag 2 and ag 3 *p* = 0.011), a modern waiting room (ANOVA *p* < 0.001; Bonferoni correction: significant differences between ag 1 and ag 3 *p* < 0.001; and ag 2 and ag 3 *p* < 0.001) or the possibility to visualize the oral situation on a screen decreases significantly (ANOVA *p* < 0.001; Bonferoni correction: significant differences between ag 1 and ag 2 *p* < 0.001; ag 1 and ag 3 *p* < 0.001, and ag 2 and ag 3 *p* < 0.001). If dentists want to welcome and treat older people, they should adapt the accessibility, infrastructure and equipment of their practice to the needs of older people in order to be able to guarantee continuous lifelong dental care regardless of the need for assistance or care.

## 1. Introduction

Dentists enjoy a high social reputation, but also struggle with the negative conditioning that has been established over centuries. This has historical reasons, as well as those to do with society and media [1]. In addition, fear and anxiety discourage many patients from utilizing the dentist [2,3,4,5]. However, there are a variety of factors that influence the utilization of dental services, either as triggers or barriers to utilization [6]. A variety and multidimensionality of reasons and behaviors exist that lead to avoidance of dental visits. Patients often have negative experiences at the dentist, but these in turn lead to different avoidance patterns and strategies [5,7].

To reduce patients’ discomfort with dentists, to enable them to have a psychologically unburdened situation during a visit, and to meet their wishes and expectations, it is essential to deal with the factors relating to satisfaction and the associated choice of the patients’ “ideal dentist”. It is well known that patient-centered care should focus on the preferences of patients as well as on the effectiveness of the clinical treatment or patients’ safety [8]. The involvment of patients in the decision-making process should be enhanced [9] to positively influence the patients’ physical and psychological outcomes by the usage of this patient-centered helthcare method [10,11]. Additionally, it has to be mentioned that there is still a lack of understanding of patient-centered care within dentistry [8].

Professional qualities often play a subordinate role for patients. More important are the dentist’s behavior [12], doctor–patient interactions and soft and psycho-social skills [12,13], such as communicativeness and informativeness [14,15,16], understanding [17], patience and respect [17,18,19], attention [12], and the ability to empower the patient with regard to the necessary treatment (i.e., discuss treatment options) [19,20,21]. What can contribute most to patient satisfaction is accepting and caring communication from the dentist [22]. Above all, the importance of mutual communication was elaborated [23]. Conversely, the literature was able to show that negative communication with a dentist leads to significantly higher levels of fear and distrust, as well as a greater likelihood of not visiting the dental office again [24]. Other authors mention the factors of trust and convenience of location as decisive in the choice of a dentist [5,21]. Additionally, an emphathic dentist [25,26] may increase the patients’ adherence to the treatments and their satisfaction, and reduce dental anxiety. The possibilty of participation in the treatment process is rated highly by patients [16]. Furthermore, the dentist should be able to listen to his or her patients. It is well known that patients have different preferences in terms of treatment decision making, but these preferences are not always met during the consultation with their dentist [27]. It is known that oral health professionals should focus more on communicating with patients who have economic problems, poor oral health, dental anxiety, or a problem-oriented visit pattern [20]. Additonally, the dentist‘s professional experience, skills and competence may play an important role for patients when choosing a dentist [20,28,29].

In today’s social media-driven world, the question arises as to whether patients use these sources of information to decide on a dentist. The influence of social media was shown to be less decisive for the choice of a surgeon than “word-of-mouth” [18,30] and the quality of the information provided during the consultation [30]. Other authors demonstrated that the influence of social media sources was also important to patients [16,31].

Concerning the dental office, factors such as reasonable waiting times for an appointment as well as conveniently timed appointments, and the attitude of the staff at the dental office seem to be important for patients [16,29,32]. Patients rated a dental office as “excellent” particularly frequently if the hygienic conditions in the office [20,33], accessibility by telephone and rapid help with urgent health problems were rated as excellent. In contrast, waiting times, unreasonable treatment costs and a lack of magazines and information material in the waiting room are not appreciated [33]. Modern equipment, pleasant furnishings and surroundings, and a good image of the practice were not as important to patients as dentists assumed [20].

It is also well known that individuals with dental/oral problems who need help with housekeeping and transportation are significantly less likely to consult a dentist than their same-age counterparts [34]. Nevertheless, selection criteria such as the loacation of the dental office, are not rated most important by patients [29].

Patient-centered dentistry should take into account all factors that motivate patients to use dental services and increase utilization, especially among seniors, for whom utilization is often reduced for various reasons [3,4,35]. It is therefore indispensable for the dentist to know what constitutes an ideal dentist–patient relationship and what factors influence utilization. Research findings indicate a significant positive relationship between service quality and patient perceptions, patient satisfaction and patient emotions. Given the increasing emphasis on patient experience and outcomes, this issue is becoming increasingly important [36].

Previous studies on the preferences of patients when chossing a dentist have been performed in different settings [13,16,18,28,29]. Therefore, a comparison or a general statement can not be made. Especially, differences between different age groups of patients have not been made in terms of awareness, selection criteria, infrastructure, and dental office equipment features. Even when patients have found their way to the dentist, it may happen that they no longer visit the dentist or switch to another one. Lucarotti and Burke were able to show that the factors that influence whether a patient switches dentists include the age of the patient, the age and gender of the dentist, and the patient’s previous visit behavior. Since some of these factors cannot be influenced directly, at least the factors within the control of the dentist and his team should be known [37]. Therefore, the aim of this study is to present factors regarding how patients become aware of a dentist, according to which criteria they select him or her and which factors in the infrastructure, equipment of dental offices, and human interactions are important for patients.

## 2. Materials and Methods

The analyzed data are part of a telephone survey of participants in the German cities of Berlin (capital of Germany), Leipzig (East-Germany) and Mainz (West-Germany) on factors influencing the choice of dentist. Participants were selected by random sampling from the relevant personal registration offices (*n* = 1400 per city). Three age groups (ag 1: 35–50 years, ag 2: 70–84 years, and ag 3: >85 years) were defined per city. All participants were interviewed by telephone by three investigators over a period of one year (2012–2013). The time required per respondent was 10–15 min. No exclusion criteria for participation were defined except that the participant had to be able to understand and answer questions in German.

The interviews were conducted with 3 interviewers who were trained on the content of the interview and how to conduct a telephone interview. Interviews were then conducted, and the interviewers did not know that they were pretest interviews. These were then discussed together with all interviewers and the principal investigator for quality assurance. The questionnaire was revised after the pre-test interviews within an expert group on the basis of the interviewers’ experience.

In the present study, part of the survey on the influence of age (ag 1 to ag 3), gender (categories: male, female) and place of residence (comparison of three major cities (Berlin as the capital of the country and one eastern (Leipzig) and one western German city (Mainz)) on the choice of dentist was evaluated. In addition to awareness and selection criteria, and gender preferences when choosing a dentist, the importance of infrastructure, dental office equipment criteria for the choice of a dentist, and human interactions were also evaluated.

For the present evaluation, multiple choice questions were used (single or multiple answers possible: questions “How did you become aware of your dentist?”, “Based on what criteria has the dentist been selected?”, possible answers: recommendation, internet, advertisement, referral, other reasons). On the other hand, 5-point Likert scales were used (criteria: very unimportant, unimportant, partly/partly, important, very important) (Questions: How important is it…) (a) that the dental office is located nearby? (b) that there is proper place to park? (c) that there is an elevator? (d) that the dental office is accessible to wheelchair users? (e) to have a modern waiting room?) to have background music in the waiting room/in the doctor’s examination room? (g) that there be a television program in the waiting room/in the doctor’s examination room? and (h) that a patient can look at his or her oral situation on a screen?.

Statistical analysis was performed using SPSS version 27.0 [38]. The statistical analysis was performed descriptively for absolute frequencies. Analysis of variance (ANOVA) with Bonferoni correction was employed to compare the different groups. The significance level was set at *p* < 0.05.

The study was approved by the competent ethics committee of the University of Leipzig (study number: 135-11-ff-18042011).

## 3. Results

For this survey, 1400 addresses per city were randomly selected (total *n* = 4200). Of these, a total of 2889 telephone numbers (72.2%) have been identified, from which a total of 1746 potential participants (60.4%) were contacted. The remaining phone numbers could not be identified. Out of all participants contacted, 1280 participants (73.3%) declined to participate in the telephone survey (reasons: lack of time, lack of interest, difficulties in communicating via telephone).

A total of 466 participants (male *n* = 187, 40.1%; female *n* = 279, 59.9%) have been included in the analysis. Almost equal proportions of equal age (Pearson Chi Square test *p* = 0.06) of the participants live in one of the three survey locations (Berlin *n* = 152, 32.6%, Leipzig *n* = 150, 32.2%, Mainz *n* = 164, 35.2%). Likewise, they belong almost equally to one of the three age groups (ag 1: *n* = 152, 32.6% (mean age: 43.3 ± 4.6 years); ag 2: *n* = 155, 33.3% (mean age: 77.3 ± 4.3 years); ag 3: *n* = 159, 34.1% (mean age: 88.9 ± 2.8 years)).

### 3.1. Awareness and Selection Criteria When Choosing a Dentist

Of all participants who answered the question regarding how they became aware of their dentist (*n* = 424, 100%), the majority (*n* = 278, 65.6%), irrespective of gender, age group and place of residence, stated that they became aware of their dentist through a recommendation. 44 participants (each 10.4%) stated advertising or other reasons (no information given by participant *n* = 21, 47.6%; proximity of dental office *n* = 12, 27.3%; dental office takeover *n* = 3, 6.8%, emergency treatment/holiday representation *n* = 3, 6.8%; previous dentist, random, opening hours, self-search, and moving to another living area each *n* = 1, 2.3%), 31 participants (7.3%) indicated the Internet, and 27 participants (6.3%) named a referral from another dentist/physician as factors in becoming aware of their dentist.

Of participants who answered the question the criteria via which selected their dentist, (*n* = 420, 100%), the majority (*n* = 238, 56.7%), irrespective of gender and age group, stated that they had chosen their dentist based on a recommendation. In Berlin and Mainz, most participants also stated that recommendations had influenced their decision (Berlin *n* = 70, 52.6%; Mainz *n* = 132, 86.8%). Only in Leipzig did 73.3% of the participants (*n* = 99) indicate other reasons as selection criteria. For about one third of the respondents, regardless of age and gender, the most important selection criterion were other reasons (*n* = 139, 33.1%, criteria: quality of work *n* = 40, 28.9%; no reasons given *n* = 38, 27.4%; proximity to the dental office *n* = 36, 25.9%; friendliness *n* = 6, 4.3%; assumption of practice *n* = 6, 4.3%; costs *n* = 5, 3.6%; sympathy/first impression *n* = 5, 3.5%; experience *n* = 2, 1.4%; opening hours *n* = 1, 0.7%) (Table 1).

Regarding the influencing factors, there were no statistical differences found between the sexes and age groups for any of the questions about awareness of dentist and selection criteria (Table 1).

There were statistically significant differences between places of residence for the question about awareness of dentist (ANOVA *p* < 0.001; Bonferoni correction: significant differences between Mainz and Berlin (*p* < 0.001), and Mainz and Leipzig (*p* < 0.001)). Additionally, there were statistically significant differences between places of residence for the question about selection criteria for a dentist (ANOVA *p* < 0.001; Bonferoni correction: significant differences between Mainz and Berlin (*p* < 0.001), Berlin and Mainz (*p*< 0.001), and Mainz and Leipzig (*p* < 0.001)) (Table 1).

### 3.2. Importance of Infrastructure as Criteria for the Choice of a Dentist

That the dental office is nearby and that there is a proper place to park is almost equally important or very important to both sexes, all age groups, and places of residence (Table 2).

Whereas male participants, participants of ag 1 and participants living in Berlin rated the availability of an elevator as unimportant compared to the other evaluation groups, almost all evaluation groups stated that it is important or very important that the dental office is accessible to wheelchair users (exception: participants from Leipzig) (Table 2).

Between the sexes, no significant differences were found for all infrastructure items (Table 2).

With increase in age, there is a statistically significant increase in the number of participants who consider the proximity of the dental office to be important or very important (proximity of the dental office is important/very important: ag 1: *n* = 97, 63.8%; ag 2: *n* = 111, 71.6%; ag 3: *n* = 130, 81.8%) (ANOVA *p* < 0.001; Bonferoni correction: significant differences between ag 1 and ag 3 *p* < 0.001). As age increases, the importance of good parking facilities (good parking facilities important or very important: ag 1: *n* = 114, 75.5%; ag 2: *n* = 110, 71.0%; ag 3: *n* = 98, 61.7% (ANOVA *p* = 0.003; Bonferoni correction: significant differences between ag 1 and ag 3 *p* = 0.004, and ag 2 and ag 3 *p* = 0.023)) decreased, while the availability of an elevator increased (elevator available important or very important: ag 1: *n* = 32, 21.2%; ag 2: *n* = 81, 52.3%; ag 3: *n* = 96, 60.3% (ANOVA *p* < 0.001; Bonferoni correction: significant differences between ag 1 and ag 2 *p* < 0.001, ag 1 and ag 3 *p* < 0.001, and ag 2 and ag 3 *p* = 0.009)), and the accessibility by wheelchair (important or very important: ag 1: *n* = 68, 44.8%; ag 2: *n* = 87, 56.2%; ag 3: *n* = 95, 59.7% (ANOVA *p* < 0.001; Bonferoni correction: significant differences between ag 1 and ag 2 *p* = 0.006; and ag 1 and ag 3 *p* < 0.001)) (Table 2).

There was a significant difference between places of residence for all items. Participants in Berlin consider the proximity of the dental office to be less important or very important compared to participants living in Leipzig or Mainz (proximity of the dental office is important/very important: Berlin: *n* = 93, 61.1%; Leipzig: *n* = 105, 70.0%, Mainz: *n* = 140, 85.4%) (ANOVA *p* < 0.001; Bonferoni correction: significant differences between Berlin and Leipzig *p* = 0.006, and Berlin and Mainz *p* < 0.001). Participants from Leipzig are least likely to consider good parking facilities important or very important compared to participants from Berlin and Mainz (good parking facilities important or very important: Berlin: *n* = 117, 77.0%; Leipzig: *n* = 84, 56.0%, Mainz: *n* = 121, 74.3%) (ANOVA *p* = 0.003; Bonferoni correction: significant differences between Berlin and Leipzig *p* = 0.003). Participants from Mainz more often found it important to have an elevator to the dental office, which should be accessible by wheelchair (ANOVA *p* = 0.013; Bonferoni correction: significant differences between Berlin and Mainz *p* = 0.004). For the item “accessibility to wheelchair users” statistically significant differences were found (ANOVA *p* < 0.001; Bonferoni correction: significant differences between Berlin and Leipzig *p* = 0.032, and Leipzig and Mainz *p* = 0.002) (Table 2).

### 3.3. Importance of Dental Office Equipment as Criteria for the Choice of a Dentist

Background music and the availability of a television program in the waiting room was unimportant for all participants, irrespective of gender, age, or place of residence. With exceptions, around one third of all participants separated by gender, age or place of residence found it unimportant, partly important, or important to have a modern waiting room at the dental office (Table 3)

Male participants, the younger age group and participants living in Mainz found it important to have a visualization of their oral situation on a screen while female participants, participants of the ag 2 and ag 3 and from Berlin and Leipzig found this aspect partly important (Table 3).

Between the sexes no significant differences were found for the items regarding dental office equipment.

The unimportance of a television (ANOVA *p* = 0.012; Bonferoni correction: significant differences between ag 1 and ag 3 *p* = 0.014; and ag 2 and ag 3 *p* = 0.011), or background music in the waiting room (ANOVA *p* < 0.001; Bonferoni correction: significant differences between ag 1 and ag 3 *p* < 0.001; and ag 2 and ag 3 *p* = 0.041), or television on the examination room (ANOVA *p* = 0.005; Bonferoni correction: significant differences between ag 1 and ag 3 *p* = 0.034; and ag 2 and ag 3 *p* = 0.007) increases with age. For participants of ag 3 a modern waiting room is less important (ANOVA *p* < 0.001; Bonferoni correction: significant differences between ag 1 and ag 3 *p* < 0.001; and ag 2 and ag 3 *p* < 0.001). The importance to visualize the oral situation on a screen decreases with increasing age (ANOVA *p* < 0.001; Bonferoni correction: significant differences between ag 1 and ag 2 *p* < 0.001; ag 1 and ag 3 *p* < 0.001, and ag 2 and ag 3 *p* < 0.001) (Table 3).

Between places of residence, no consensus emerged for the importance of a modern waiting room (ANOVA *p* = 0.005; Bonferoni correction: significant differences between Berlin and Leipzig *p* = 0.02). For all other items significant differences were found between the places of residence (Table 3).

Data on patients’ perceptions about personal characteristics and soft skills of an ideal dentist can be found in part II: Patients’ preferences when choosing a dentist—Part II: Personal characteristics and soft skills (under consideration).

## 4. Discussion

To date, some information on patients’ expectations of an ideal dentist and how to establish a “feel-good factor”, especially for old and very old patients, can be found in the literature [39]. Most literature deals with the dentist‘s personality, behavior, and communication skills as described above, whereas information is lacking on how patients of different ages or from different locations become aware of a dentist, their selection criteria, and the influence of dental office equipment. For this reason, the results evaluated in this study cannot directly be compared with those of other studies. 

### 4.1. Study Limitations

The number of participants in the telephone survey who could be included in the evaluation is high. This can be explained by the possibility of follow-up recruitment. Nevertheless, the overall response rate is around two thirds, which is acceptable for a telephone survey. Due to changes in culture, marketing and the entire field of telecommunications, response rates for telephone surveys are declining [40]. Especially among younger people, mobile telephones are primarily used, whose numbers often cannot be found in the telephone directories where landlines are kept. Thus, a bias with regard to the selection of study participants cannot be excluded.

Additionally, there may be a bias with regard to the oldest age group. Within this age group (>85 years of age) more independently living people may have contributed to the telephone survey. It is possible that people who are dependent on care are underrepresented in all age groups—especially in the oldest age group where a higher prevalence of people in need of care is typical. Therefore, there may be a bias with regard to the answers regarding mobility and accessibility.

In the present study, consistent interviewer training before the field phase ensured that research bias was very low. The interviewers kept a log, from which difficulties in implementation could be inferred. These protocols show that, when a person was recruited for the interview, there were no difficulties in carrying it through to the end. Certainly, experience with telephone interviews and a training period beforehand influence the outcome, as described in the literature [41,42].

In selecting the cities, and by omitting inclusion and exclusion criteria to the greatest extent possible (inclusion of people 35 years and older) and evaluating by age and gender, the authors attempted to obtain the broadest possible variance among participants while also obtaining a targeted evaluation, e.g., by age. Nevertheless, the statements obtained should be interpreted with caution, as bias cannot be ruled out. It is possible that, on the one hand, mainly people who regularly visit the dentist took part in the survey. On the other hand, people with a low utilization rate did not participate, although their answers are particularly interesting (barriers to utilization). 

Furthermore, it is possible that questions were not understood as well over the telephone, or that, due to the lack of visual support during answer selection: a) predominantly first- or last-order answer choices were selected and b) participants chose extreme answers less often for the Likert scales. Furthermore, it cannot be excluded that participants answered within expected norms of social desirability. 

### 4.2. Interpretation of Results

When evaluating the results, it should be kept in mind that it is not the response of the individual participant but a tendency in general that is important for the interpretation. Therefore, attention should be paid to the importance or unimportance of the respective factors. For this purpose, one has to combine the responses of the individual participants (e.g., important/very important).

Despite the current wide range of multimedia options for patient advertising, the main factor that attracts patients’ attention to their dentist and by which they select him or her appears to be recommendation. This was shown in the present study irrespective of age, gender, and place of residence (expect for the city of Leipzig), as well as in previous studies [18]. A possible explanation for this could be that information passed on in direct human interaction is rated as more trustworthy by people than anonymous advertisements. The unimportance of new technologies, e.g., the internet, for becoming aware of a dentist increases with increasing age. This contrasts with the findings of Kim et al. [16], who showed that both traditional (recommendation from family, friends, etc.) and modern sources of information (Internet, social media, etc.) are important in getting patients’ attention. The difference between this study and the present work, however, is that modern media and technologies were not of interest, especially for older people, in becoming aware of and choosing their dentist. Dentists facing an older patient clientele due to demographic change should keep this in mind, despite the ever-increasing popularity of the use of social media channels by dentists [43] and younger patients [31]. Ungureanu et al. demonstrated that the dentist’s competence was the most important aspect when choosing a dentist, followed by recommendations, and the quality of the dental service [18]. The willingness to recommend one’s own dentist to friends increases if the dentist listens to the patients and takes enough time, and also depends on the outcome of the dental treatment from the patients’ point of view [33]. Therefore, dentists should not only rely on these new ways to acquire patients, although a high number believe that social media marketing is more effective than traditional acquisition of patients [43]. Additionally, a referral from professionals was the most likely reason to influence the patient‘s choice, as reported in another study by Gray et al. [21]. Alsaeed et al. demonstrated that the dentist’s reputation, clinical rank and cost of treatment were important factors for patients when choosing their dentist, but the study participants were young patients with a mean age of 30.5 ± 11.6 years [31]. Therefore, this study is not comparable with the results described here.

Other authors were able to show that, regardless of regional, gender and socioeconomic differences, the same influencing factors were evident: patient care and attention, efficient pain control, and the importance of their well-being to the dentist, among others, are very important to them. These factors reflect the behavior and personal skills of the dentist who devotes time and attention to the patient [12]. However, in the present study, practice factors were investigated. Since the proximity of the dental office and the availability of parking facilities are equally important or very important to both sexes, but increases with increasing age, and is more important to people in Berlin, the underlying causes can only be speculated on. Especially in large cities and for younger people, the proximity of the practice and the availability of parking spaces could be a less significant point, as local public transport is well developed, and they are able to use it. Interestingly, with the increase in age, there is a statistically significant increase in the number of participants who consider the proximity of the dental office, good parking facilities (except age group 3—older than 85 years of age), the availability of an elevator, and accessibility by wheelchair are more important or very important. This can be well explained by increasing immobility in old age, with an increase in chronic diseases and the need for care. Interestingly, good parking is less important or very important to the oldest participants. One explanation could be that the oldest participants are more often frail and may be dependent on ambulance transport or driven by relatives. Therefore, the parking possibility at the practice could play a less important role for them personally since it does not directly affect them. It also shows that these factors should be considered when planning a senior-friendly dental office, and presumably have an influence on the utilization behavior of elderly patients. Other studies have also shown that patients value the convenience of the location of the dental office [5,21].

With the increase in age, a modern waiting room is less important, the unimportance of background music and television in the waiting room increases, and the importance to of visualizing the oral situation on a screen decreases. Here, too, the factors that make up a senior-friendly dental office are clear [39]. Above all, the lack of interest in background music and television in old age can be well explained by the hearing loss that often increases with age. It is therefore often difficult for older patients to follow conversations while other sources of noise are present (music, noise caused by the work of dental assistants in the background, etc.). This in turn is likely to influence patient management, patient satisfaction and ultimately the treatment outcome. Other authors also point out that the use of modern technology, e.g., the use of electronic patient records, can lead to communication with the patient suffering. Some patients even rated this as frustrating and distracting [44]. If modern media are considered, it could be shown that the validity of online content is often secondary to the strength of the network via which it is shared. In addition, it may be that online advocacy could be more effective in enabling emotional connections with peers rather than maintaining the accuracy of the information [45]. Up-to-date equipment, a pleasant decor and surroundings and good practice image were reported to be less important to most patients, while most dentists believe that these factors are essential [20].

When comparing the cities of Leipzig and Mainz, one has to take into account that there are more old people aged 60 years of age or older living in Leipzig than in Mainz. The old age quotient, which is the ratio of persons of retirement age (e.g., 65 years and older) to 100 persons of working age, is higher in Leipzig (old age quotient 49.3 (2020) [46]) than in Mainz (old age quotient 35.4 (2019) [47]). Nevertheless, e.g., a nearby location of the dental office and dental office accessibility by wheelchair are more important to people in Mainz than in Leipzig. Additionally, music or television in the waiting room/examination room is less important to people from Mainz. The differences could be due to the historical developments of the two regions (Mainz in West Germany, Leipzig in the former GDR). It is possible that different socialization has led to supposedly modern aspects, such as music and television in the dentist’s office, being more favored in Leipzig as a sign of modernity due to social desirability. With regard to the structural conditions, it can be assumed that here, too, a reason lies in the historical development. The city of Leipzig has been structurally expanded and renewed in recent decades, which is often associated with the expansion of public transport and the construction of parking spaces due to building regulations. In Mainz, on the other hand, which is also known for its historic old town, construction projects are probably more difficult to implement. The limited space might have led to a lower availability of parking spaces and public transport connections, which could explain the increased value of these aspects for people from Mainz.

Since no literature exist which deals with infrastructure or dental office equipment as factors or aspects for patients when choosing their ideal dentist, no comparison with the results of this study is possible. At the same time, however, this also shows that the importance of accessibility to dental care in the locality of the dental office is not at the forefront of science. However, this must be questioned in view of an increasingly older society. Especially, older patients whose need for assistance and care is increasing, but who still live and are cared for at home, can be cut off from dental treatment and care by inadequate accessibility to a dental practice. In view of the fact that mobile dental care is not established across the board in the outpatient home setting, this must be viewed as critical. This could be one of the reasons why the utilization of a dentist decreases with increase in age and concomitant multimorbidity [3,4]. Here, it is necessary to clarify causes and establish structures to counteract this process—the loss of older people in dental practice [4].

## 5. Conclusions

In the selection of the dentist and the selection criteria applied, there are hardly any differences between the sexes, the age, or the place of residence of the patient. It is interesting to note, however, that as age increases factors such as infrastructure and dental office equipment become increasingly important and influence the choice of dentist. In view of demographic change, it therefore makes sense for dentists to adapt their dental offices to the needs of older people in terms of accessibility, infrastructure, and office equipment to enable and ensure lifelong dental care regardless of the need for assistance or care.

## Figures and Tables

**Table 1:** Awareness and selection criteria in the choice of dentist presented separately by gender (female *n* = 279, male *n* = 187), age group (ag 1 *n* = 152, ag 2 *n* = 155, ag 3 *n* = 159) and place of residence (Berlin *n* = 152, Leipzig *n* = 150, Mainz *n* = 164). (Total (all participants) *n* = 466; *n*/%—number/percent, ag—age groups; bold values in column *p* indicate statistical significance with a significance level of *p* < 0.05, *p* = ANOVA with Bonferoni correction for age group and residence of living/without Bonferoni post-hoc test for sex, * indicates the differences between two cities.

	Total	Sex			Age Group				Residence of Living				R^2^
	All	Female	Male	*p*	ag 1	ag 2	ag 3	*p*	Berlin (B) (*n*/%)	Leipzig (L) (*n*/%)	Mainz (M) (*n*/%)	*p*	
					35–50 Yrs.	70–84 Yrs.	85+ Yrs.						
	(*n*/%)	(*n*/%)	(*n*/%)		(*n*/%)	(*n*/%)	(*n*/%)						
How did you become aware of your dentist?													
	*n* = 424	*n* = 251	*n* = 173	0.060	*n* = 143	*n* = 149	*n* = 132	0.587	*n* = 134	*n* = 135	*n* = 155	ANOVA **< 0.001** Bonferoni M * B **< 0.001** M * L **< 0.001**	0.103
Recommendation	278/65.6	174/69.3	104/60.1		94/65.7	96/64.4	88/66.7		79/59.0	63/46.7	136/87.7		
Internet	31/7.3	14/5.6	17/9.8		26/18.2	4/2.7	1/0.8		11/8.2	18/13.3	2/1.3		
Advertisement	44/10.4	24/9.6	20/11.6		10/7.0	18/12.1	16/12.1		26/19.4	15/11.1	3/1.9		
Referral	27/6.3	14/5.6	13/7.5		1/0.7	16/10.7	10/7.6		5/3.7	18/13.3	4/2.6		
Other reasons	44/10.4	25/10.0	19/10.2		12/8.4	15/10.1	17/12.9		13/9.7	21/15.6	10/6.5		
Based on what criteria has the dentist been selected?													
	*n* = 420	*n* = 250	*n* = 170	0.932	*n* = 142	*n* = 147	*n* = 131	0.649	*n* = 133	*n* = 135	*n* = 152	ANOVA **< 0.001** Bonferoni B * M **< 0.001** B * L **< 0.001** L * M **< 0.001**	0.316
Recommendation	238/56.7	146/58.4	92/54.1		74/52.1	88/59.9	76/58.0		70/52.6	36/26.7	132/86.8		
Internet	11/2.6	6/2.4	5/2.9		8/5.6	2/1.4	1/0.8		10/7.5	0/0	1/0.7		
Advertisement	25/6.0	13/5.2	12/7.1		6/4.2	10/6.8	9/6.9		22/16.5	0/0	3/2.0		
Referral	7/1.6	4/1.6	3/1.8		0/0	3/2.0	4/3.1		3/2.3	0/0	4/2.6		
Other reasons	139/33.1	81/32.4	58/34.1		54/38.0	44/29.9	41/31.3		28/21.1	99/73.3	12/7.9		

**Table 2:** Importance of infrastructure as criteria for the choice of a dentist presented separately by gender (female *n* = 279, male *n* = 187), age group (ag 1 *n* = 152, ag 2 *n* = 155, ag 3 *n* = 159) and place of residence (Berlin *n* = 152, Leipzig *n* = 150, Mainz *n* = 164). (Total (all participants) *n* = 466; *n*/%—number/percent, ag—age groups; bold values in column *p* indicate statistical significance with a significance level of *p* < 0.05, *p* = ANOVA with Bonferoni correction for age group and residence of living/without Bonferoni post-hoc test for sex, * indicates the differences between two cities or age groups.

	Total	Sex			Age Group				Place of Residenc				R^2^
	All	Female	Male	*p*	ag 1	ag 2	ag 3	*p*	Berlin (B) (*n*/%)	Leipzig (L) (*n*/%)	Mainz (M) (*n*/%)	*p*	
					35–50 Yrs.	70–84 Yrs.	85 + Yrs.						
	(*n*/%)	(*n*/%)	(*n*/%)		(*n*/%)	(*n*/%)	(*n*/%)						
How important is it that the dental office is located nearby?													
very unimportant	3/0.7	1/0.4	2/1.1	0.056	1/0.7	2/1.3	0/0	ANOVA **< 0.001** Bonferoni ag 1 * ag 3 **< 0.001**	3/2.0	0/0	0/0	ANOVA **< 0.001** Bonferoni B * L **0.006** B * M **< 0.001**	0.141
unimportant	35/7.5	18/6.5	17/9.1		20/13.2	9/5.8	6/3.8		18/11.8	10/6.7	7/4.3		
partly/partly	90/19.3	48/17.2	42/22.5		34/22.4	33/21.3	23/14.5		38/25.0	35/23.3	17/10.4		
important	229/49.1	137/49.1	92/49.2		79/52.0	78/50.3	72/45.3		68/44.7	63/42.0	98/59.8		
very important	109/23.4	75/26.9	34/18.2		18/11.8	33/21.3	58/36.5		25/16.4	42/28.0	42/25.6		
How important is it that there is proper place to park?													
	*n* = 465	*n* = 278	*n* = 187	0.798	*n* = 150	*n* = 155	*n* = 160	ANOVA **0.003** Bonferoni ag 1 * ag 3 **0.004** ag 2 * ag 3 **0.023**	*n* = 152	*n* = 150	*n* = 163	ANOVA **0.003** Bonferoni B * L **0.003**	0.108
very unimportant	8/1.8	4/1.4	4/2.1		2/1.3	2/1.3	4/2.5		1/0.7	6/4.0	1/0.6		
unimportant	67/14.4	46/16.5	21/11.2		11/7.3	16/10.3	40/25.2		13/8.6	29/19.3	25/15.3		
partly/partly	68/14.6	36/12.9	32/17.1		24/15.9	27/17.4	17/10.7		21/13.8	31/20.7	16/9.8		
important	234/50.3	131/47.0	103/55.1		84/55.6	79/51.0	71/44.7		88/57.9	53/35.3	93/57.1		
very important	88/18.9	61/21.9	27/14.4		30/19.9	31/20.0	27/17.0		29/19.1	31/20.7	28/17.2		
How important is it that there is an elevator?													
	*n* = 465	*n* = 279	*n* = 186	0.085	*n* = 150	*n* = 155	*n* = 160	ANOVA **< 0.001** Bonferoni ag 1 * ag 2 **< 0.001** ag 1 * ag 3 **< 0.001** ag 2 * ag 3 **0.009**	*n* = 152	*n* = 150	*n* = 163	ANOVA **0.013** Bonferoni B * M **0.004**	0.248
very unimportant	23/4.9	12/4.3	11/5.9		16/10.6	4/2.6	3/1.9		9/5.9	9/6.0	5/3.1		
unimportant	119/25.6	61/21.9	58/31.2		68/45.0	34/21.9	17/10.7		45/29.6	41/27.3	33/20.2		
partly/partly	114/24.5	68/24.4	46/24.7		35/23.2	36/23.29	43/27.0		42/27.6	42/28.0	20/18.4		
important	150/32.3	100/35.8	50/26.9		26/17.2	64/41.3	60/37.7		39/25.7	33/22.0	78/47.9		
very important	59/12.7	38/13.6	21/11.3		6/4.0	17/11.0	36/22.6		17/11.2	25/16.7	17/10.4		
How important is it that the dental office is accessible to wheelchair users?													
very unimportant	10/2.1	5/1.8	5/2.7	0.435	7/4.6	3/1.9	0/0	ANOVA **< 0.001** Bonferoni ag 1 * ag 2 **0.006** ag 1 * ag 3 **< 0.001**	5/3.3	5/3.3	0/0	ANOVA **< 0.001** Bonferoni B * L **0.032** L * M **0.002**	0.151
unimportant	72/15.5	42/15.1	30/16.0		39/25.7	18/11.6	15/9.4		21/13.8	30/20.0	21/12.8		
partly/partly	134/28.8	75/26.9	59/31.6		38/25.0	47/30.3	49/30.8		38/25.0	53/35.3	43/26.2		
important	213/45.7	132/47.3	81/43.3		58/38.2	79/51.0	76/47.8		73/48.0	51/34.0	89/54.3		
very important	37/7.9	25/9.0	12/6.4		10/6.6	8/5.2	19/11.9		15/9.9	11/7.3	11/6.7		

**Table 3:** Importance of dental office equipment, and human interactions as criteria for the choice of a dentist presented separately by gender (female *n* = 279, male *n* = 187), age group (ag 1 *n* = 152, ag 2 *n* = 155, ag 3 *n* = 159) and place of residence (Berlin *n* = 152, Leipzig *n* = 150, Mainz *n* = 164). (Total (all participants) *n* = 466; *n*/%—number/percent, ag—age group; bold values in column *p* indicate statistical significance with a significance level of *p* < 0.05, *p* = ANOVA with Bonferoni correction for age group and residence of living/without Bonferoni post-hoc test for sex, * indicates the differences between two cities or age groups.

	Total	Sex			Age Group				Residence of Living				R^2^
	All	Female	Male	*p*	ag 1	ag 2	ag 3	*p*	Berlin	Leipzig	Mainz	*p*	
					35–50 Yrs.	70–84 Yrs.	85 + Yrs.		(B)	(L)	(M)		
	(*n*/%)	(*n*/%)	(*n*/%)		(*n*/%)	(*n*/%)	(*n*/%)		(*n*/%)	(*n*/%)	(*n*/%)		
How important is it to have a modern waiting room?													
very unimportant	16/3.4	9/3.2	7/3.7	0.299	3/2.0	2/1.3	11/6.9	ANOVA **< 0.001** Bonferoni ag 1 * ag 3 **< 0.001** ag 2 * ag 3 **< 0.001**	8/5.3	4/2.7	4/2.4	ANOVA **0.005** Bonferoni B * L **0.020**	0.111
unimportant	154/33.0	99/35.5	55/29.4		36/23.7	45/29.0	73/45.9		40/26.3	49/32.7	65/39.6		
partly/partly	142/30.5	83/29.7	59/31.6		56/36.8	44/28.4	42/26.4		43/28.3	71/48.0	27/16.5		
important	144/30.9	84/30.1	60/32.1		52/34.2	60/38.7	32/20.1		55/36.2	24/16.0	65/39.6		
very important	10/2.2	4/1.4	6/3.2		5/3.3	4/2.6	1/0.6		6/3.9	1/0.7	3/1.8		
How important is it to have background music in the waiting room?													
very unimportant	40/8.6	19/6.8	21/11.2	0.830	19/12.5	8/5.2	13/8.2	0.317	15/9.9	12/8.0	13/7.9	ANOVA **< 0.001** Bonferoni B * M **< 0.001** L * M **< 0.001**	0.094
unimportant	211/45.3	134/48.0	77/41.2		59/38.8	71/45.8	81/50.9		52/34.2	54/36.0	105/64.0		
partly/partly	124/26.6	73/26.2	51/27.3		34/22.4	48/31.0	42/26.4		47/30.9	53/35.3	24/14.6		
important	87/18.7	52/18.6	35/18.7		38/25.0	26/16.8	23/14.5		36/23.7	29/19.3	22/13.4		
very important	4/0.8	1/0.4	3/1.6		2/1.3	2/1.3	0/0		2/1.3	2/1.3	0/0		
How important is it that there is a television program in the waiting room?													
very unimportant	82/17.6	50/17.9	32/17.1	0.389	26/17.1	21/13.5	35/22.0	ANOVA **0.012** Bonferoni ag 1 * ag 3 **0.014** ag 2 * ag 3 **0.011**	49/32.2	16/10.7	17/10.4	ANOVA **< 0.001** Bonferoni B * L **0.002** L * M **0.006**	0.104
unimportant	247/53.0	151/54.1	96/51.3		75/49.3	80/51.6	92/57.9		60/39.5	67/44.7	120/73.2		
partly/partly	91/19.5	55 8 19.7	36/19.3		32/21.1	38/24.5	21/13.2		23/15.1	54/36.0	14/8.5		
important	44/9.4	23/8.2	21/11.2		17/11.2	16/10.3	11/6.9		19/12.5	12/8.0	13/7.9		
very important	2/0.5	0/0	2/1.1		2/1.3	0/0	0/0		1/0.7	1/0.7	0/0		
How important is it to have background music in the doctor’s examination room?													
very unimportant	80/17.2	47/16.8	33/17.6	0.526	24/15.8	18/11.6	38/23.9	ANOVA **< 0.001** Bonferoni ag 1 * ag 3 **< 0.001** ag 2 * ag 3 **0.041**	52/34.2	11/7.3	17/10.4	ANOVA **< 0.001** Bonferoni B * L **< 0.001** L * M **< 0.001**	0.117
unimportant	205/44.0	123/44.1	82/43.9		52/34.2	77/49.7	76/47.8		39/25.7	61/40.7	105/64.0		
partly/partly	112/24.0	67/24.0	45/24.1		41/27.0	40/25.8	31/19.5		48/31.6	43/28.7	21/12.8		
important	64/13.7	40/14.3	24/12.8		31/20.4	19/12.3	14/8.8		12/7.9	31/20.7	21/12.8		
very important	5/1.1	2/0.7	3/1.6		4/2.6	1/0.6	0/0		1/0.7	4/2.7	0/0		
How important is it that there is a television program in the doctor’s examination room													
very unimportant	118/25.3	69/24.7	49/26.2	0.905	39/25.7	30/19.4	49/30.8	ANOVA **0.005** Bonferoni ag 1 * ag 3 **0.034** ag 2 * ag 3 **0.007**	84/55.3	16/10.7	18/11.0	ANOVA **< 0.001** Bonferoni B * L **< 0.001** B * M **< 0.001**	0.234
unimportant	242/51.9	146/52.3	96/51.3		75/49.3	81/52.3	86/54.1		54/35.5	75/50.0	113/68.9		
partly/partly	73/15.7	45/16.1	28/15.0		20/13.2	34/21.9	19/11.9		12/7.9	46/30.7	15/9.1		
important	30/6.4	18/6.5	12/6.4		15/9.9	10/6.5	5/3.1		1/0.7	11/7.3	18/11.0		
very important	3/0,7	1/0.4	2/1.1		3/2.0	0/0	0/0		1/0.7	2/1.3	0/0		
How important is it that a patient can look at his or her oral situation on a screen?													
very unimportant	42/9.0	24/8.6	18/9.6	0.757	6/3.9	8/5.2	28/17.6	ANOVA **< 0.001** Bonferoni ag 1 * ag 2 **< 0.001** ag 1 * ag 3 **< 0.001** ag 2 * ag 3 **< 0.001**	37/24.3	5/3.3	0/0	ANOVA **< 0.001** Bonferoni B * L **< 0.001** B * M **< 0.001**	0.256
unimportant	108/23.2	69/24.7	39/20.9		21/13.8	37/23.9	50/31.4		37/24.3	26/17.3	45/27.4		
partly/partly	155/33.3	95/34.1	60/32.1		37/24.3	64/41.3	54/34.0		45/29.6	64/42.7	46/28.0		
important	143/30.7	82/29.4	61/32.6		73/48.0	45/29.0	25/15.7		27/17.8	49/32.7	67/40.9		
very important	18/3.8	9/3.2	9/4.8		15/9.9	1/0.6	2/1.3		6/3.9	6/4.0	6/3.7		

## Data Availability

The data presented in this study are available from the corresponding author upon request. The data are not publicly available due to ethical reasons.

## References

[1] Wolf C.A., Ramseier C.A. (2012). The image of the dentist. Part 1: Results of a literature search. Schweiz Mon. Zahnmed.

[2] Pohjola V., Lahti S., Vehkalahti M.M., Tolvanen M., Hausen H. (2007). Association between Dental Fear and Dental Attendance among Adults in Finland. Acta Odontol. Scand..

[3] Nitschke I., Stillhart A., Kunze J. (2015). Utilization of Dental Services in Old Age. Swiss Dent. J..

[4] Nitschke I., Hahnel S., Jockusch J. (2021). Health-Related Social and Ethical Considerations towards the Utilization of Dental Medical Services by Seniors: Influencing and Protective Factors, Vulnerability, Resilience and Sense of Coherence. Int. J. Env. Res. Public Health.

[5] Mittal R., Wong M.L., Koh G.C.-H., Ong D.L.S., Lee Y.H., Tan M.N., Allen P.F. (2019). Factors Affecting Dental Service Utilisation among Older Singaporeans Eligible for Subsidized Dental Care—A Qualitative Study. BMC Public Health.

[6] Fox C. (2010). Evidence Summary: What Do We Know from Qualitative Research about People’s Care-Seeking about Oral Health?. Br. Dent. J..

[7] Gragoll I., Schumann L., Neubauer M., Westphal C., Lang H. (2021). Healthcare Avoidance: A Qualitative Study of Dental Care Avoidance in Germany in Terms of Emergent Behaviours and Characteristics. BMC Oral Health.

[8] Mills I., Frost J., Cooper C., Moles D.R., Kay E. (2014). Patient-Centred Care in General Dental Practice—A Systematic Review of the Literature. BMC Oral Health.

[9] Scambler S., Delgado M., Asimakopoulou K. (2016). Defining Patient-Centred Care in Dentistry? A Systematic Review of the Dental Literature. Br. Dent. J..

[10] Rathert C., Wyrwich M.D., Boren S.A. (2013). Patient-Centered Care and Outcomes: A Systematic Review of the Literature. Med. Care Res. Rev..

[11] World Health Organization (2015). People-Centred and Integrated Health Services: An Overview of the Evidence: Interim Report.

[12] Holt V.P., McHugh K. (1997). Factors Influencing Patient Loyalty to Dentist and Dental Practice. Br. Dent. J..

[13] Lamprecht R., Struppek J., Heydecke G., Reissmann D.R. (2020). Patients’ Criteria for Choosing a Dentist: Comparison between a University-Based Setting and Private Dental Practices. J. Oral Rehabil..

[14] Lahti S., Tuutti H., Hausen H., Kaariainen R. (1992). Dentist and Patient Opinions about the Ideal Dentist and Patient -Developing a Compact Questionnaire. Commun. Dent. Oral Epidemiol..

[15] Lahti S., Tuutti H., Hausen H., Kaarianen R. (1996). Patients’ Expectations of an Ideal Dentist and Their Views Concerning the Dentist They Visited: Do the Views Conform to the Expectations and What Determines How Well They Conform?. Commun. Dent. Oral Epidemiol..

[16] Kim M.J., Damiano P.C., Hand J., Denehy G.E., Cobb D.S., Qian F. (2012). Consumers’ Choice of Dentists: How and Why People Choose Dental School Faculty Members as Their Oral Health Care Providers. J. Dent. Educ..

[17] Gheorghiu I.-M., Scărlătescu S., Mitran L., Nicola G., Perlea P., Iliescu A.A., Mitran M. (2021). Importanţa Relaţiei Dintre Medicul Dentist Şi Pacient Pentru Un Tratament Stomatologic de Succes. ORL.ro.

[18] Ungureanu M.-I., Mocean F. (2015). What Do Patients Take into Account When They Choose Their Dentist? Implications for Quality Improvement. Patient Prefer. Adherence.

[19] Sbaraini A., Carter S.M., Evans R.W., Blinkhorn A. (2012). Experiences of Dental Care: What Do Patients Value?. BMC Health Serv. Res..

[20] Burke L., Croucher R. (1996). Criteria of Good Dental Practice Generated by General Dental Practitioners and Patients. Int. Dent. J..

[21] Gray L., McNeill L., Yi W., Zvonereva A., Brunton P., Mei L. (2021). The “Business” of Dentistry: Consumers’ (Patients’) Criteria in the Selection and Evaluation of Dental Services. PLoS ONE.

[22] Corah N.L., O’Shea R.M., Bissell G.D. (1985). The Dentist-Patient Relationship: Perceptions by Patients of Dentist Behavior in Relation to Satisfaction and Anxiety. J. Am. Dent. Assoc..

[23] Lahti S., Verkasalo M., Hausen H., Tuutti H. (1996). Ideal Role Behaviours as Seen by Dentists and Patients Themselves and by Their Role Partners: Do They Differ?. Community Dent. Oral Epidemiol..

[24] Fico A.E., Lagoe C. (2018). Patients’ Perspectives of Oral Healthcare Providers’ Communication: Considering the Impact of Message Source and Content. Health Commun..

[25] Bendapudi N.M., Berry L.L., Frey K.A., Parish J.T., Rayburn W.L. (2006). Patients’ Perspectives on Ideal Physician Behaviors. Mayo Clin. Proc..

[26] Jones L.M., Huggins T.J. (2014). Empathy in the Dentist-Patient Relationship: Review and Application. NZ Dent. J..

[27] Chapple H., Shah S., Caress A.-L., Kay E.J. (2003). Exploring Dental Patients’ Preferred Roles in Treatment Decision-Making—A Novel Approach. Br. Dent. J..

[28] Swami V., McClelland A., Bedi R., Furnham A. (2011). The Influence of Practitioner Nationality, Experience, and Sex in Shaping Patient Preferences for Dentists. Int. Dent. J..

[29] Iqbal M., Jameel A., Girach M. (2014). Factors Affecting Patients’ Choice of Dental Services. Pak. Oral Dent. J..

[30] Chapon M.-P., Ghabi A., Choufani C., Caubere A., Moynot J.-C., Versier G., Wein F., Barbier O. (2021). How Do Patients Choose Their Surgeon? Example of Anterior Cruciate Ligament Reconstruction. Orthop. Traumatol. Surg. Res..

[31] Alsaeed S., Alghurairi N., Almutairi L., Alossimi A., Bin Fadhl A., Abahussain S. (2021). Factors That Affect Saudi Population Preferences Toward Their Dentist. Patient Prefer. Adherence.

[32] Sever I., Verbič M., Sever E.K. (2018). Valuing the Delivery of Dental Care: Heterogeneity in Patients’ Preferences and Willingness-to-Pay for Dental Care Attributes. J. Dent..

[33] Klingenberg A., Walther W., Dörfer C., Szecsenyi J. (2008). Wie bewerten Patienten ihre zahnärztliche Versorgung? Ergebnisse einer schriftlichen Befragung von Patienten bei niedergelassenen Zahnärzten. Gesundheitswesen.

[34] Marchini L., Reynolds J.C., Caplan D.J., Sasser S., Russell C. (2020). Predictors of Having a Dentist among Older Adults in Iowa. Community Dent. Oral Epidemiol..

[35] Jockusch J., Hopfenmüller W., Nitschke I. (2021). Influence of Cognitive Impairment and Dementia on Oral Health and the Utilization of Dental Services: Findings of the Oral Health, Bite Force and Dementia Study (OrBiD). BMC Oral Health.

[36] Sag I., Zengul F.D., Landry A.Y. (2018). Patient Perceptions of Servicescape in Healthcare: A Systematic Review of the Literature. J. Healthc. Manag..

[37] Lucarotti P.S.K., Burke F.J.T. (2015). Factors Influencing Patients’ Continuing Attendance at a given Dentist. Br. Dent. J..

[38] IBM (2021). SPSS Statistics for Windows.

[39] Nitschke I., Bleiel D., Ludwig E., Wefers K.-P., Nitschke S. (2018). Die seniorengerechte Praxis. Der Gerostomatologische Wohlfühlfaktor. Z. Seniroenzahnmedizin.

[40] O’Toole J., Sinclair M., Leder K. (2008). Maximising Response Rates in Household Telephone Surveys. BMC Med. Res. Methodol..

[41] Chen X.W., Shafei M.N., Abdullah J.M., Musa K.I. (2019). Reliability of Telephone Interview for Assessment of Long-Term Stroke Outcomes: Evidence from Interrater Analysis. Neuroepidemiology.

[42] Ahern T., Gardner A., Gardner G., Middleton S., Della P. (2013). Development and Interrater Reliability Testing of a Telephone Interview Training Programme for Australian Nurse Interviewers. Nurse Educ. Today.

[43] Parmar N., Dong L., Eisingerich A.B. (2018). Connecting With Your Dentist on Facebook: Patients’ and Dentists’ Attitudes Towards Social Media Usage in Dentistry. J. Med. Internet Res..

[44] Asan O., Ye Z., Acharya A. (2013). Dental Care Providers & apos; and Patients & apos; Perceptions of the Effect of Health Information Technology in the Dental Care Setting. J. Am. Dent. Assoc..

[45] Seymour B., Yang H., Getman R., Barrow J., Kalenderian E. (2016). Patient-Centered Communication: Exploring the Dentist’s Role in the Era of e-Patients and Health 2.0. J. Dent. Educ..

[46] Statistisches Landesamt Freistaat Sachsen 7. Regionalisierte Bevölkerungsvorausberechnung für den Freistaat Sachsen 2019 Bis 2035. https://www.bevoelkerungsmonitor.sachsen.de/download/RBV%20Kreise/rbv-landkreisinfo_landkreis-leipzig.pdf.

[47] Statistisches Landesamt Rheinland-Pfalz (2020). Statistisches Jahrbuch. https://www.statistik.rlp.de/fileadmin/dokumente/jahrbuch/Jahrbuch_2020_Kapitel_2_-_Bevoelkerung.pdf.

